# Targeting Gut Microbiota for the Prevention and Management of Diabetes Mellitus by Dietary Natural Products

**DOI:** 10.3390/foods8100440

**Published:** 2019-09-25

**Authors:** Bang-Yan Li, Xiao-Yu Xu, Ren-You Gan, Quan-Cai Sun, Jin-Ming Meng, Ao Shang, Qian-Qian Mao, Hua-Bin Li

**Affiliations:** 1 Guangdong Provincial Key Laboratory of Food, Nutrition and Health, Department of Nutrition, School of Public Health, Sun Yat-sen University, Guangzhou 510080, China; liby35@mail2.sysu.edu.cn (B.-Y.L.); xuxy53@mail2.sysu.edu.cn (X.-Y.X.); mengjm@mail2.sysu.edu.cn (J.-M.M.); shangao@mail2.sysu.edu.cn (A.S.); maoqq@mail2.sysu.edu.cn (Q.-Q.M.); 2 Institute of Urban Agriculture, Chinese Academy of Agricultural Sciences, Chengdu 610213, China; 3 Department of Food Science & Technology, School of Agriculture and Biology, Shanghai Jiao Tong University, Shanghai 200240, China; 4 School of Food and Biological Engineering, Jiangsu University, Zhenjiang 212013, China; sqctp8@ujs.edu.cn

**Keywords:** gut microbiota, natural products, diabetes mellitus, complications, mechanisms

## Abstract

Diabetes mellitus is one of the biggest public health concerns worldwide, which includes type 1 diabetes mellitus, type 2 diabetes mellitus, gestational diabetes mellitus, and other rare forms of diabetes mellitus. Accumulating evidence has revealed that intestinal microbiota is closely associated with the initiation and progression of diabetes mellitus. In addition, various dietary natural products and their bioactive components have exhibited anti-diabetic activity by modulating intestinal microbiota. This review addresses the relationship between gut microbiota and diabetes mellitus, and discusses the effects of natural products on diabetes mellitus and its complications by modulating gut microbiota, with special attention paid to the mechanisms of action. It is hoped that this review paper can be helpful for better understanding of the relationships among natural products, gut microbiota, and diabetes mellitus.

## 1. Introduction

Over the past 20 years, the prevalence of diabetes mellitus (DM) and its complications has rapidly increased across the world, which poses a serious threat to global health [1,2,3]. The international diabetes federation (IDF) estimated that one in eleven adults aged 20 to 79 (415 million) have DM globally in 2015, and predicts that the number can rise to 642 million by 2040 [4]. DM is a complex metabolic disorder characterized with the functional impairment or a lack of insulin-producing β-cells, alone or in combination with insulin resistance [5]. DM has several types, including type 1 diabetes mellitus (T1DM), type 2 diabetes mellitus (T2DM), gestational diabetes mellitus (GDM), and other rare forms of diabetes mellitus [6,7]. T1DM is an autoimmune disease that is associated with the aberrant immune responses to specific β-cell autoantigens, which causes the reduction of insulin [8,9,10,11]. T2DM is due to insulin resistance in the target tissue and a relative lack of insulin secreted by islet β-cells, and is mainly affected by the combination of genetic susceptibility and lifestyle factors [12,13,14,15]. GDM is a kind of diabetes during pregnancy due to the increased severity of insulin resistance and an impairment of the compensatory increase in insulin secretion [16,17,18]. Other rare forms of diabetes mellitus, such as maturity-onset of diabetes of the young (MODY) resulted from mutations in a single gene. In addition, maternally inherited diabetes with deafness (MIDD) is caused by mitochondrial mutations, and rare forms resulted from insulin gene mutations [19,20]. In this review paper, only T2DM and T1DM will be discussed, because T2DM and T1DM are the main types of diabetes mellitus, accounting for 90%–95% and 5%–10%, respectively [4,21,22]. In addition, T2DM can induce various complications, which contributes to high morbidity and mortality, such as diabetic cardiovascular diseases, nephropathy, retinopathy, neuropathy, and erectile dysfunctions [23,24]. Furthermore, many studies have shown that some natural products and their bioactive components could prevent and manage diabetes mellitus and its complications by several possible mechanisms, such as enhancing insulin action, ameliorating insulin resistance, activating insulin signaling pathway, protecting islet β-cells, scavenging free radicals, decreasing inflammation, and modulating gut microbiota [25,26,27,28,29]. In recent years, the role of gut microbiota in the prevention and management of diabetes mellitus and its complications has been a hot topic in research [14,30,31,32]. Special attention will be paid to the role of gut microbiota in this paper.

Gut microbiota has been recently reported to be involved in the pathogenesis of several diseases, such as obesity [33,34,35,36,37], liver diseases [38,39,40,41], cardiovascular diseases [42,43,44], cancer [45,46], and DM [47,48,49,50]. The alteration of intestinal microbiota and the production of metabolites have been demonstrated to play a critical role in the initiation and development of DM [51,52,53,54]. Some bacteria like *Saccharomyces boulardii* Biocodex, *Bifidobacterium animalis* subsp. lactis 420, *Lactobacillus casei* CCFM419, and *Clostridium butyricum* CGMCC0313.1 have been shown to ameliorate DM [55,56,57,58,59]. However, it was reported that some bacteria, like *Bacteroides* and *Candida albicans*, promoted the incidence and progression of T2DM in animal studies [60,61]. On the other hand, natural products have attracted wide attention in recent years due to their diverse bioactivities, such as cardiovascular protective, hepatoprotective, anti-cancer, anti-obesity, and anti-diabetic effects [25,36,62,63,64,65,66,67,68,69,70,71]. Furthermore, natural products are also important factors in regulating gut microbiota [72,73,74]. A variety of dietary natural products and their bioactive components have shown anti-diabetic effects by regulating gut microbiota composition and abundance, the change of gut permeability, the production of short-chain fatty acids (SCFAs), the decrease of lipopolysaccharides (LPS), and the inhibition of inflammation [58,75,76,77,78,79,80]. In addition, natural products can be effective adjuvant agents in the therapy of T2DM with lower cost and less side effects [81,82]. In this review paper, we first address the relationship between gut microbiota and T1DM as well as T2DM based on animal and epidemiological studies, and then discuss the effects of natural products on T2DM and its complications via modulation of gut microbiota, based on the literature from animal and clinical studies in the last five years (2015–2019).

## 2. The Association Between Gut Microbiota and DM Based on Animal and Epidemiological Studies

Many animal studies have revealed that the gut microbiota plays an important role in the incidence and development of T1DM as well as T2DM [54,75,83,84]. In addition, increasing investigations have found that gut microbiota is closely associated with T1DM and T2DM patients [85,86,87], and they will be discussed in detail below.

### 2.1. The Association between Gut Microbiota and T1DM

The animal studies have shown that T1DM has been associated with the composition of the gut microbiota [88]. For example, the abundance of *Proteobacteria* showed a more marked change than that of the predominant phyla, such as *Firmicutes* and *Bacteroidetes* compared with the healthy control in the rat model of T1DM induced by streptozotocin [89]. In another study, the gut microbiota from non-obese diabetic (NOD) mice without myeloid differentiation primary response gene 88 (MyD88) was transferred to wild-type NOD mice, which reduced the intensity of insulitis and delayed the onset of autoimmune diabetes in recipients, by decreasing the amount of *Lactobacillaceae* and increasing the number of *Lachnospiraceae* and *Clostridiaceae* [59]. Moreover, the deficiency of TIR-domain-containing adapter-inducing interferon-β in NOD mice could prevent diabetes via the change in the gut microbiota, such as the proportion of *Sutterella*, *Rikenella,* and *Turicibacter* species [90]. In addition, the mouse β-defensin 14 (mBD14) in pancreatic endocrine cells in NOD mice could prevent autoimmune diabetes, while the microbiota dysbiosis could induce the deficiency of the pancreatic expression of mBD14, which results in the higher incidence of T1DM [91].

The relationship between gut microbiota and T1DM has been summarized in a review paper published in 2015 [54]. In addition, the epidemiological studies have reported that some intestinal microbiota like phylum *Bacteroidetes*, genus *Clostridium*, *Bacteroides*, and *Veillonella* as well as *Candida albicans* increased in T1DM patients, while some other gut microbiota like phylum *Actinobacteria* and *Firmicutes*, genus *Lactobacillus*, *Bifidobacterium*, and *Faecalibacterium* as well as the ratio of *Firmicutes* to *Bacteroidetes* decreased in T1DM patients [92,93,94,95,96].

### 2.2. The Association between Gut Microbiota and T2DM

The animal studies have found that there have been prominent differences in the composition of gut microbiota between T2DM animal models and control subjects. For example, an *in vivo* study found that the diabetic *db/db* mice had higher abundance of phyla *Firmicutes*, *Proteobacteria*, and *Fibrobacteres* [97]. Additionally, the streptozotocin-induced T2DM mice showed an increase in *Brevibacterium*, *Corynebacterium*, and *Facklamia* compared with wild type mice [98]. A decrease in the diversity of gut microbiota and a lack of butyrate-producing bacteria were observed in T2DM cats [99]. The level of serum fructosamine was inversely associated with the abundance of *Prevotellaceae*, and was positively associated with the abundance of *Enterobacteriaceae*. In addition, the transfer of *Lachnospiraceae* (strain AJ110941) from the feces of hyperglycemic obese mice to germ-free *ob/ob* mice contributed to the development of T2DM in *ob/ob* mice. The colonization of *Lachnospiraceae* could induce an increase in the level of fasting blood glucose (FBG) and a decrease in the level of plasma insulin as well as homeostasis model assessment-β (HOMA-β). The decrease in HOMA-β values indicated the dysfunction of pancreatic β-cells [100].

The epidemiological studies have also investigated the relationship between gut microbiota and T2DM patients [101,102,103,104]. For example, a case-control study has shown that the proportions of phylum *Firmicutes* and class *Clostridia* as well as the ratio of *Firmicutes* to *Bacteroidetes* decreased, whereas the abundance of class *Betaproteobacteria* increased in T2DM patients [101]. In addition, the concentration of *Faecalibacterium prausnitzii* and the abundance of genus *Blautia* was reduced in T2DM patients [102,103]. Moreover, a case-control study demonstrated that T2DM and obese patients had a lower quantity of *Lactobacillus*, especially its subgroups (*L. acidophilus*, *L. plantarum*, and *L. reuteri*) in comparison with controls [104]. 

Overall, several animal studies have revealed that the intestinal microbiota play an important role in T1DM and T2DM. In addition, many epidemiological investigations have demonstrated the closed association between gut microbiota and T2DM (Table 1). Furthermore, there are few prospective studies on the relationship between gut microbiota and DM, which should be further investigated in the future.

## 3. The Relationships among Natural Products, Gut Microbiota, and T2DM as well as T1DM Based on Animal Studies

Some natural products, such as vegetables, fruits, dietary fibers, and medicinal plants, have shown potential preventive effects against T2DM and T1DM as well as diabetic complications with mechanisms of action, at least partly, via the modulation of gut microbiota [80,105,106,107,108].

### 3.1. Diabetes Mellitus

The extracts of vegetables and their bioactive components have been demonstrated to alleviate T2DM by modulating the gut microbiota [75,79,80]. Capsaicin could improve the glucose homeostasis and insulin tolerance in obese diabetic *ob/ob* mice by increasing the production of SCFAs and modulating gut microbiota. It could increase the ratio of *Firmicutes* to *Bacteroidetes* and the quantity of *Roseburia*, and reduce the quantities of *Bacteroides* and *Parabacteroides* at the genus level, which could decrease the levels of pro-inflammatory cytokines, such as TNF-α and IL-6 [79]. Additionally, pumpkin polysaccharide alleviated the T2DM in mice fed with a high-fat diet (HFD). Its antidiabetic effects were also related to the increase in SCFAs production and selective enhancement of some bacteria, such as *Bacteroidetes*, *Prevotella,* and *Deltaproteobacteria* [75]. Moreover, the carrot juice fermented with *Lactobacillus rhamnosus* GG was demonstrated to ameliorate T2DM in rats via the increase in SCFAs in the cecum and the change in the composition and abundance of gut microbiota, such as *Oscillibacter* and *Akkermansia* [80]. In addition, the anti-diabetic activity of *Momordica charantia* (bitter melon) was enhanced by the fermentation of *Lactobacillus*, which could induce an increase in the abundance of *Bacteroides caecigallinarwn*, *Bacteroides thetaiotaomicron*, *Prevotella loescheii*, *Prevotella oralis*, and *Prevotella melaninogenica* as well as the level of SCFAs [109].

It has also been reported that the extracts of several fruits and their bioactive components could improve T2DM via gut microbiota [105,110,111]. The phlorizin in many fruits could competitively inhibit the sodium-glucose symporters and regulate the level of blood glucose by decreasing the level of serum LPS and insulin resistance, and increasing the level of SCFAs, especially butyric acid. It could also increase the quantity of *Akkermansia muciniphila* and *Prevotella*, which modulates the gut microbial community structure [110]. In addition, polysaccharides from the mulberry fruit have shown protective effects against T2DM via gut microbiota in *db*/*db* mice, and the treatment with polysaccharides could enrich the functional bacteria, such as *Bacteroidales, Lactobacillus*, *Allobaculum*, *Bacteroides*, and *Akkermansia* [105]. The extracts from cinnamon bark were reported to improve glucose tolerance and insulin resistance by reducing the abundance of genus *Peptococcus*, and the extracts from grape pomace could reduce *Desulfovibrio* and *Lactococcus,* and increase *Allobaculum* and *Roseburia* in diabetic mice [111].

The inulin was also found to alleviate different stages of T2DM in diabetic mice by modulating gut microbiota. It increased the relative abundance of *Cyanobacteria* and *Bacteroides*, and reduced the relative abundance of *Deferribacteres* and *Tenericutes* [112]. In another study, the oral administration with inulin-type fructan could reduce the FBG level, increase the glucagon-like peptide-1 (GLP-1) level, and alleviate glucose intolerance as well as blood lipid in T2DM rats induced by HFD and streptozotocin [108]. The treatment with inulin also increased the level of GLP-1 and improved the gut microbiota dysbiosis by enriching probiotic bacteria *Lactobacillus* and SCFAs-producing bacteria *Lachnospiraceae*, *Phascolarctobacterium*, and *Bacteroides*. Furthermore, the supplementation of the long-chain inulin-type fructan fibers from chicory root to female NOD mice could delay the progression of T1DM via the modulation of gut microbiota. This fiber could increase the ratio of *Firmicutes* to *Bacteroidetes* and the abundance of *Ruminococcaceae* and *Lactobacilli*, which might contribute to the increase in the expression of tight junction proteins occludin and claudin-2 and an antidiabetogenic effect [106]. 

Some traditional Chinese medicine have been found to be effective in preventing and treating T2DM. The administration of ethanol extract of *Atractylodis macrocephalae* rhizoma improved the glucose metabolism by regulating the gut microbiota in diabetic *db*/*db* mice, and it could increase the abundance of *Bacteroides thetaiotaomicron* and *Methanobrevibacter smithii* [113]. In addition, the extract of *Alpinia oxyphylla* Miq. had protective effects against diabetes in T2DM *db*/*db* mice. It decreased the blood glucose levels by modulating the intestinal microbiota composition, which increased the abundance of *Akkermansia* and decreased *Helicobacter* [114]. The water extract of *Potentilla discolor* Bunge could alleviate diabetes, which might be related to the change in the gut microbiota. The treatment reduced the relative abundance of *Proteobacteria* in T2DM mice induced by HFD and streptozotocin [77]. It was related to an increase in levels of fecal acetic acid, butyric acid, and their specific receptors including the expression of G-protein-coupled receptor 41 (GPR41) and G-protein-coupled receptor 43 (GPR43), which increased the insulin sensitivity and decreased the fat accumulation. Additionally, the expressions of toll-like receptor-4 (TLR4), MyD88, nuclear factor-kappa B (NF-κB), and gut mucosal tight junction proteins (caudin-3 and occludin) were decreased, which could reduce the gut permeability and inflammation. In addition, the oral administration of total saponins and polysaccharides in *Polygonatum kingianum* could prevent T2DM by regulating the gut microbiota [115]. The treatment improved micro-ecology in the gut by reducing the abundance of *Bacteroidetes* and *Proteobacteria*, and increasing *Firmicutes*. Furthermore, the baicalein showed anti-diabetic effects on diabetic rats, which reduced the levels of blood glucose, LPS, and insulin resistance. The treatment of baicalein could regulate the gut microbiota by increasing the relative abundance of *Bacteroides* and *Bacteroidales* S24-7 [78]. It could also increase the production of SCFAs as well as the thickness of the gut mucus layer. 

Overall, many studies have focused on the effects of different natural products, such as vegetables, fruits, dietary fibers, and medicinal plants, on T2DM by regulating gut microbiota and improving microecology in the gut. On the other hand, there are few studies about the effect of natural products on T1DM by modulating gut microbiota. In the future, more studies are needed to investigate the effects of different natural products and bioactive components on T1DM via gut microbiota-related mechanisms. 

### 3.2. Diabetic Complications

Long-term diabetes increases the likelihood of its complications like diabetic erectile dysfunctions, nephropathy, cardiovascular diseases, retinopathy, and neuropathy [116,117]. The gut microbiota has also been found to have a close relationship with the development of the diabetic complications. In the rat models with T2DM erectile dysfunction, there was a reduction in the relative abundance of beneficial bacteria like *Allobaculum*, *Bifidobacterium*, *Eubacterium*, and *Anaerotruncus*. In addition, the relative abundance of opportunistic pathogens increased, such as *Enterococcus*, *Corynebacterium*, *Aerococcus*, and *Facklamia* [118]. Some natural products have been reported to have protective effects on diabetic nephropathy or kidney injury in diabetic mice by modulating the gut microbiota balance [107,119]. For example, the polyphenolic extract of *Dendrobium loddigesii* could improve the symptoms of diabetes and complications in diabetic *db*/*db* mice. These effects were likely related to the improvement of the intestinal flora balance, with an increase in the relative abundance of *Prevotella* and *Akkermansia*, and the reduction in the relative abundance of S24-7/*Rikenella*/*Escherichia coli* [119]. Additionally, the treatment with the water-ethanolic extract of green macroalgae *Enteromorpha prolifera* reduced the inflammation in the liver and kidney by significantly increasing the abundance of *Lachnospiraceae* and *Alisties* as well as regulating the insulin signaling pathway in T2DM mice induced by HFD and high sucrose diet (HSD) and streptozotocin [107]. 

To sum up, several studies have demonstrated that the gut microbiota has a tight association with diabetic complications. The increase or decrease in different types of gut microbiota could exert different effects on the progression of diabetic complications. Moreover, several natural products have showed potential efficacy on the prevention and management against diabetic complications, and these effects were mainly associated with the modulation of gut microbiota composition and abundance, regulation of the insulin signaling pathway, and inhibition of inflammation.

**Figure 1:**
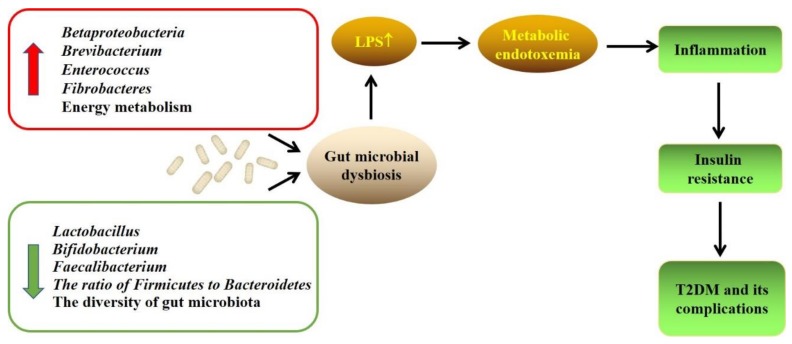
The association among gut microbiota, T2DM, and its complications. The changes of gut microbiota caused the increase in LPS, which could cause inflammation and insulin resistance. It indicates that gut microbiota would play an important role in the initiation and development of T2DM and its complications. Abbreviations: LPS, lipopolysaccharides; T2DM, type 2 diabetes mellitus.

**Figure 2:**
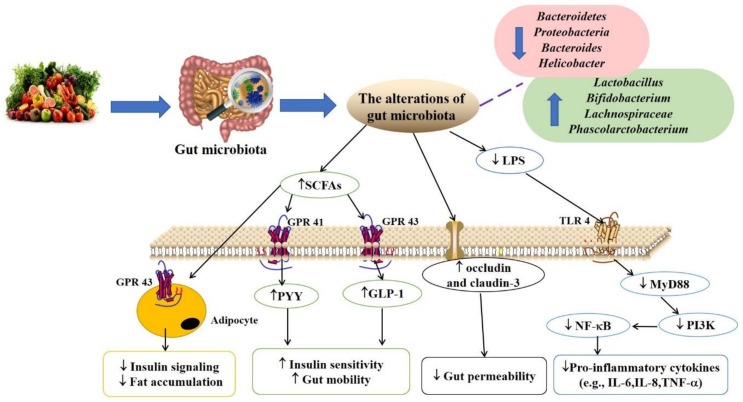
The relationship among natural products, gut microbiota, T2DM, and its complications. Some natural products and their bioactive components alleviate T2DM by changing the composition and abundance of gut microbiota. It decreases the permeability of gut and the level of LPS, increases the production of SCFAs, and inhibits the inflammation. The arrows out of the box indicates the direction of action, and the up arrows in the box mean the upregulation or activation, and the down arrows in the box mean the downregulation or inhibition. Abbreviations: LPS, lipopolysaccharides. SCFAs, short-chain fatty acids. T2DM, type 2 diabetes mellitus. GPR41/43, G-protein-coupled receptor41/43. TLR4, toll-like receptor-4. GLP-1, glucagon-like peptide-1. PYY, peptide YY. MyD88, myeloid differentiation primary response gene 88. PI3K, phosphatidylinositol-3-kinase. NF-κB, nuclear factor-kappa B. IL-6, interleukin 6. IL-8, interleukin 8. TNF-α, tumor necrosis factor-α.

Lastly, the relationship among gut microbiota, T2DM, and its complications is shown in Figure 1, and the association among natural products, gut microbiota, T2DM, and its complications is given in Table 2 and Figure 2.

## 4. The Relationships among Natural Products, Gut Microbiota, and T2DM Based on Clinical Studies

Several clinical trials have evaluated the protective effects of natural products on T2DM via gut microbiota [120,121]. A double-blind, randomized, controlled clinical trial (RCT) involving 60 patients with T2DM found that the supplementation of 10 g/day inulin powder promoted the gut health by increasing the proportion of *Akkermansia muciniphila* [120]. Moreover, an RCT study found that the fiber-rich macrobiotic Ma-Pi 2 diet could regulate gut microbiome dysbiosis in obese T2DM patients by increasing the gut microbiota ecosystem diversity, recovering the SCFAs-producing bacteria community, such as *Roseburia*, *Lachnospira*, *Faecalibacterium*, *Bacteroides,* and *Akkermansia*, and inhibiting the increase of the pro-inflammatory group, such as *Collinsella* and *Streptococcus* [122]. Furthermore, an RCT study including 100 patients with T2DM reported that a Chinese herbal formula could ameliorate T2DM and improved the glucose and lipid homeostasis by increasing the abundance of *Faecalibacterium* spp. [116]. Another RCT study observed that the treatment with Gegen Qinlian decoction, which is a type of Chinese herbal formula, at the moderate and high doses, significantly reduced the mean alterations in adjusted FBG and glycated hemoglobin A1c (HbA1c) levels by enriching the beneficial bacteria, such as *Faecalibacterium prausnitzii* [121].

To sum up, several clinical trials demonstrate that natural products can ameliorate T2DM by regulating gut microbiota (Table 3). Some natural products, such as inulin and medicine plants, are able to enrich certain beneficial bacteria in T2DM patients, such as *Akkermansia muciniphila*, *Faecalibacterium,* and *Lachnospira*. 

## 5. Conclusions

In conclusion, the relationship among diabetes mellitus (including T1DM and T2DM), gut microbiota, and natural products has been summarized and discussed. Animal and epidemiological studies found significant differences in intestinal microbiota composition and abundance between diabetic patients and healthy controls. The abundance of *Bifidobacterium* and *Lactobacillus* as well as the ratio of *Firmicutes* to *Bacteroidetes* decreased, while the abundance of *Bacteroidetes* and *Proteobacteria* increased in T1DM and T2DM patients. However, the consequences were not always consistent due to other factors like types of diets and progression of diseases. In addition, animal studies have demonstrated that gut microbiota is one of the most important factors for diabetes initiation and development. Several natural products possessing prebiotic effects like fruits, vegetables, and medicinal plants, have been found to ameliorate T2DM by modulating gut microbiota composition and abundance, reducing the gut permeability, increasing the production of SCFAs, decreasing the level of LPS, and inhibiting the inflammation. Moreover, clinical trials have further confirmed that several natural products are effective in preventing and treating T2DM, with regulation of gut microbiota as one of the potential mechanisms. In the future, it is necessary to explore the effects of more natural products and their bioactive components on DM via gut microbiota-related mechanisms. In addition, more well-designed clinical trials on natural products and their various bioactive compounds should be carried out to verify their effects on T2DM and its complications by regulating gut microbiota. Furthermore, functional foods based on natural products can be researched and developed by targeting gut microbiota for the prevention and management of T2DM. On the other hand, present studies mainly focus on modulating the action of natural products and their bioactive components on gut microbiota for preventing and managing T2DM. However, seldom studies have been carried out about the metabolism of natural products and their bioactive components by gut microbiota, even though the metabolites could directly play a role in the prevention and management of DM or, in return, could modulate gut microbiota. Thus, metabolisms of natural products and their bioactive components by gut microbiota should be widely studied in the future for the prevention and management of DM. 

## Figures and Tables

**Table 1:** The epidemiological studies on the association between gut microbiota and T2DM.

Study Types	Participants	Alterations in Gut Microbiota Composition	Reference
Case-control study	Adults with T2DM (*n* = 18) Healthy male adults (*n* = 18)	↓ The proportions of phylum *Firmicutes* and class *Clostridia*, and the ratios of *Firmicutes* to *Bacteroidetes* ↑ The proportion of *betaproteobacteria*	[101]
Case-control study	Patients with T2DM (*n* = 18) Healthy individuals (*n* = 18)	↓ The concentration of *Faecalibacterium prausnitzii*	[102]
Case-control study	Patients with T2DM (*n* = 10) Healthy individuals (*n* = 12)	↓ The abundance of genus *Blautia*	[103]
Case-control study	Patients with T2DM (*n* = 100) Healthy individuals (*n* = 100)	↓ The counts of *Lactobacillus* sp. (*L. acidophilus*, *L. plantarum*, and *L. reuteri*) and *Bifidobacterium*	[104]

Abbreviations: T2DM, Type 2 diabetes mellitus. ↓, Decrease. ↑, Increase.

**Table 2:** The animal studies of natural products on DM and its complications by modulating gut microbiota.

Natural Products	Disease	Study Types	Models	Effects	Mechanisms	Reference
Long-chain inulin-type fructans fibers	T1DM	*In vivo*	NOD diabetic mice	Promoting modulatory T-cell responses. Delaying the development of T1DM.	↑ The ratio of *Firmicutes* to *Bacteroidetes* ↑ The abundance of *Ruminococcaceae* and *Lactobacilli* ↑ The production of SCFAs ↑ The expression of tight junction proteins occludin and claudin-2	[106]
Capsaicin	T2DM	*In vivo*	Obese T2DM *ob*/*ob* mice	Inhibiting the levels of FBG and insulin. Improving the glucose homeostasis and insulin tolerance.	↑ The ratio of *Firmicutes* to *Bacteroidetes* ↑ The abundance of *Roseburia* ↑ The production of butyrate ↓ The quantities of *Bacteroides* and *Parabacteroides* ↓ The levels of TNF-α and IL-6	[79]
Pumpkin polysaccharide	T2DM	*In vivo*	T2DM rats	Improving insulin tolerance. Decreasing the levels of glucose, TC, LDL-C. Increasing the level of HDL-C.	↑ The quantities of some bacteria, such as *Bacteroidetes*, *Prevotella*, *Deltaproteobacteria*, *Oscillospira*, *Veillonellaceae*, *Phascolarctobacterium*, *Sutterella*, and *Bilophila* ↑ The production of SCFAs	[75]
Fermented carrot juice	T2DM	*In vivo*	T2DM rats	Regulating the levels of blood glucose and insulin as well as the morphology of the pancreas and kidney.	↑ The quantities of *Christensenellaceae*, *Oscillibacter*, *Ruminococcaceae*, *Lachnospiraceae*, and *Akkermansia* ↑ The level of SCFAs	[80]
Fermented *Momordica charantia* juice	T2DM	*In vivo*	T2DM rats induced by HFD and STZ	Relieving the hyperglycemia, hyperinsulinemia, and hyperlipidemia.	↑ The abundance of *Bacteroides caecigallinarwn*, *Bacteroides thetaiotaomicron*, *Prevotella loescheii*, *Prevotella oralis*, and *Prevotella melaninogenica* ↑ The concentrations of acetic acid, propionic acid, and butyric acid	[109]
Phlorizin	T2DM	*In vivo*	T2DM *db*/*db* mice	Reducing insulin resistance. Regulating the level of blood glucose.	↑ The abundance of *Akkermansia muciniphila* and *Prevotella* ↑ The gut microbial diversity ↑ The production of butyric acid ↓ The level of LPS	[110]
Mulberry fruit polysaccharide	T2DM	*In vivo*	T2DM *db*/*db* mice	Improving glucose tolerance. Increasing the level of HDL-C. Decreasing the levels of TC TG, LDL-C, and FFA. Inhibiting body weight gain.	↑ The abundance of *Bacteroidales*, *Lactobacillus*, *Allobaculum*, *Bacteroides*, and *Akkermansia* ↓ The gut microbial diversity	[105]
Cinnamon bark and grape pomace extracts	T2DM	*In vivo*	T2DM C57BL/6J mice induced by HFD	Improving glucose tolerance and insulin resistance. Decreasing fat mass gain and adipose tissue inflammation.	↑ The abundances of *Allobaculum* and *Roseburia,* ↑ The expression of tight junction proteins. ↓ The abundance of genus *Peptococcus*, *Desulfovibrio*, and *Lactococcus*	[111]
Inulin	T2DM	*In vivo*	Mice	Decreasing the levels of FBG and glycated hemoglobin, and body weight.	↑ The relative abundance of *Cyanobacteria* and *Bacteroides* ↓ The relative abundance of *Deferribacteres* and *Tenericutes*	[112]
Inulin-type fructan	T2DM	*In vivo*	T2DM rats induced by HFD/STZ	Reducing the levels of FBG, IL-6, and alleviated glucose intolerance.	↑ The abundance of *Lactobacillus* and SCFAs-producing bacteria, such as *Lachnospiraceae*, *Phascolarctobacterium*, and *Bacteroides* ↑ The level of GLP-1	[108]
*Atractylodis macrocephalae* Rhizoma ethanol extract	T2DM	*In vivo*	T2DM *db*/*db* mice	Decreasing the levels of blood glucose, TG, TC, endotoxin, and IL-10	↑ The abundance of *Bacteroides thetaiotaomicron* and *Methanobrevibacter smithii*	[113]
*Alpinia oxyphylla* Miq. extract	T2DM	*In vivo*	T2DM *db*/*db* mice	Improving glycemic control and renal function	↑ The ratio of *Bacteroidetes* to *Firmicutes* and ↑ The abundance of *Akkermansia* ↓ The abundance of *Helicobacter*	[114]
*Potentilla discolor* Bunge water extract	T2DM	*In vivo*	T2DM C57BL/6J mice induced by HFD and STZ	Decreasing the level of pro-inflammatory cytokines. Improving inflammation.	↑ The concentrations of fecal acetic acid butyric acid and their specific receptors including the expression of GPR41 and GPR43 ↓ The ratio of *Firmicutes* to *Bacteroidetes* and ↓ The abundance of *Proteobacteria* ↓ The expression of gut mucosal tight junction proteins (Claudin3, ZO-1, and Occludin), TLR4, MyD88, and NF-κB	[77]
*Polygonatum kingianu*	T2DM	*In vivo*	T2DM SD rats induced by HFD and STZ	Increasing the level of fasting insulin. Preventing the increase of FBG. Improving the intestinal microecology.	↑ The abundance of *Firmicutes* ↓The abundances of *Bacteroidetes* and *Proteobacteria*	[115]
Baicalein	T2DM	*In vivo*	Diabetic rats induced by HFD, HSD, and STZ	Decreasing the level of blood glucose. Improved inflammation and lipid metabolism.	↑ The relative abundances of *Bacteroides* and *Bacteroidales* S24-7 ↑ The production of SCFAs ↑ The thickness of the gut mucus layer	[78]
*Dendrobium loddigesii* polyphenols extract	Diabetic nephropathy	*In vivo*	Diabetic nephropathy *db*/*db* mice	Improving diabetic nephropathy. Decreasing blood glucose level. Increasing the level of insulin.	↑ The relative abundance of *Prevotella*/*Akkermansia* ↓ The relative abundance of S24-7/*Rikenella*/*Escherichia coli*	[119]
Green seaweed *Enteromorpha prolifera* flavonoids	T2DM	*In vivo*	T2DM mice induced by HFD and HSD and STZ	Decreasing the level of FBG. Improving glucose tolerance. Reducing inflammation. Preventing liver and kidney injury.	↑ The abundances of *Lachnospiraceae* and *Alisties* ↑ The IRS1/PI3K/AKT pathway ↓ The JNK1/2 insulin pathway in liver	[107]

Abbreviations: DM, diabetes mellitus. T1DM, type 1 diabetes mellitus. T2DM, type 2 diabetes mellitus. NOD mice, non-obese diabetic mice. HFD, high-fat diet. HSD, high sucrose diet. STZ, streptozotocin. LPS, lipopolysaccharides. FBG, fasting blood glucose. GLP-1, glucagon-like peptide-1. TG, triglyceride. HOMA-IR, homeostasis model assessment of insulin resistance. TC, total cholesterol. FFA, free fatty acid. SCFAs, short-chain fatty acids. IL-6, interleukin 6.IL-10, interleukin 10. TLR4, toll-like receptor-4. TNF-α, tumor necrosis factor-α. MyD88, myeloid differentiation primary response gene 88. HDL-C, high-density lipoprotein cholesterol. LDL-C, low-density lipoprotein cholesterol. NF-κB, nuclear factor-kappa B. GPR41/43, G-protein-coupled receptor 41/43. IRS1, insulin receptor substrate 1. PI3K, phosphatidylinositol-3-kinase. AKT, protein kinase B. JNK, Jun N-terminal kinase.

**Table 3:** The clinical trials of natural products on T2DM by modulating gut microbiota.

Natural Products	Study Types	Participants	Dose	Duration	Results	Reference
Inulin	RCT	T2DM patients (*n* = 60)	10 g/day	N/A	↑ The proportion of *Akkermansia muciniphila*	[120]
Macrobiotic Ma-Pi 2 diet enriched fiber	RCT	Obese T2DM patients (*n* = 56)	N/A	N/A	↑ Gut microbiota ecosystem diversity ↑ SCFA-producing bacteria, such as *Faecalibacterium*, *Roseburia*, *Lachnospira*, *Bacteroides* and *Akkermansia* ↓ Pro-inflammatory bacteria, such as *Collinsella* and *Streptococcus*	[122]
Chinese herbal formula	RCT	T2DM patients (*n* = 100)	N/A	12 weeks	↑ The abundance of *Faecalibacterium spp* ↓ Hyperglycemia and hyperlipidemia	[123]
Ge gen Qin lian decoction	RCT	T2DM patients (*n* = 187)	N/A	12 weeks	↑ The number of *Faecalibacterium prausnitzii* ↓ The mean changes of FBG and HbA1c levels	[121]

Abbreviations: N/A, not available. RCT, randomized control clinical trial. FBG, fasting blood glucose. T2DM, type 2 diabetes mellitus. HbA1c, hemoglobin A1c. SCFA, short-chain fatty acids.
